# Agent-based modeling of erosion and sloughing during growth of *Pseudomonas aeruginosa* biofilms

**DOI:** 10.1038/s41522-026-01052-1

**Published:** 2026-06-22

**Authors:** Ryan Bournes, Suzie Hingley-Wilson, Bing Guo, Cayla Harris, Umar Abubacar, Mark Chambers, Roman Bauer

**Affiliations:** 1 Computer Science Research Centre, University of Surrey, Guildford, UK; 2 School of Biosciences, University of Surrey, Guildford, UK; 3 School of Sustainability, Civil and Environmental Engineering, University of Surrey, Guildford, UK

**Keywords:** Computational biology and bioinformatics, Engineering, Mathematics and computing, Microbiology

## Abstract

*Pseudomonas aeruginosa* is a prevalent healthcare pathogen. It forms biofilms, which significantly increase antibiotic resistance, escalating the burden on healthcare systems. While global biofilm phenomena are observable experimentally, understanding local behaviours of bacteria and their environment, and how these factors drive biofilm formation, is challenging. Agent-based modelling (ABM) offers a computational solution to simulate these local behaviours and resulting large-scale phenomena. ABMs can simulate physical mechanisms like erosion and sloughing but often lack the ability to capture them during biofilm growth and validate them experimentally. We present a three-dimensional ABM of *P. aeruginosa* biofilms growing under constant flow. This model is the first of its kind to accurately capture the change in physical shape of biofilms growing under constant flow, as well as capture erosion and sloughing as natural byproducts of local bacteria interactions. The model is validated both qualitatively and quantitatively against experimental culture results, providing a general framework that can be adapted to simulate different environments or other biofilm-forming species.

## Introduction

Biofilms are microbial communities that secrete an adhesive extracellular polymeric substance (EPS), allowing cells to attach to surfaces and resist harsh conditions^1^. Biofilms are found in a variety of environments and industries and can be problematic, such as causing infections in healthcare^2^, or helpful, such as removing ammonia from wastewater^3^. As of 2019, biofilms have been reported to have a global economic significance of almost $4 trillion^4^. The EPS barrier helps pathogens withstand antibiotics and aseptic techniques, even promoting antibiotic resistance mutations^5^. Effective treatment is crucial for reducing healthcare costs and patient mortality.

*Pseudomonas aeruginosa* is a motile, gram-negative bacterium often linked to infection and medical equipment contamination^6^. Primarily an opportunistic nosocomial pathogen, it infects immunocompromised patients, causing airway, urinary tract, burn, and wound infections. Its genetic versatility enables high adaptability^7^. *P. aeruginosa* biofilms can harbour other pathogens^8^ and limit antibiotic diffusion through the EPS matrix, promoting intrinsic tolerance^9^. The virulence of *P. aeruginosa* is linked to its quorum-sensing capabilities that trigger phenotypic changes in response to the density and nature of surrounding bacteria^10^. Due to these traits, in 2017 the World Health Organisation listed *P. aeruginosa* as one of the most life-threatening pathogens and called for more research effort in the development of antibiotics to tackle it^11^.

As the adaptability of *P. aeruginosa* allows it to survive in different environments, research efforts have focused on preventing the growth and spread of *P. aeruginosa* biofilms^8,12,13^, which are also sources of dissemination. Bacteria detach via erosion (frequent release of single cells or small clumps^14^), sloughing (less frequent release of large clumps that alter biofilm shape^15^), and dispersion (voluntary exit triggered by environmental shifts and gene expression changes^16^). For *P. aeruginosa*, dispersion occurs through secretions of quorum-sensing-mediated surfactant called rhamnolipids which prevent the bacteria from sticking to the EPS matrix, allowing it to swim out of the biofilm^17^. These methods determine bacterial spread and survival.

Observing local bacterial interactions in situ is difficult without disrupting the biofilm. Thus, biofilm research turns to mathematical modelling to gain a better understanding of the local interactions of bacteria in their natural state^1^. Agent-based modelling (ABM) uses autonomous agents defined by rules to simulate global phenomena from local interactions^18^, making it ideal for modelling biofilms. The most recent major advancement in ABMs of biofilms is the introduction of physical phenomena such as agent forces and fluid dynamics^19–21^, giving ABM biofilms a realistic shape and structure. Previous ABMs have managed to simulate biofilms in both still media^22^ and fluid flow^21^. However, the most sophisticated ABMs simulate erosion and sloughing using biofilms grown in a static medium which is then subjected to shear stress^19,21^. This can be uncharacteristic of many biofilms seen in nature^23,24^, particularly *P. aeruginosa* biofilms which grow under constant flow in environments like catheters^25^ and plumbing networks^26^. Combining growth and detachment modelling under flow, validated by experiments, is necessary to accurately capture their behaviour.

This study introduces a three-dimensional ABM of a *P. aeruginosa* biofilm growing under constant fluid flow that captures erosion, sloughing, and dispersion throughout its life cycle. The model integrates an ABM for bacterial behaviours and collision physics with a finite volume method for fluid dynamics. A multi-scale approach handles long-timescale growth and short-timescale collision/detachment. Biofilm structure, detachment rates, and detached clump size distributions are compared both qualitatively (varying EPS production/flow speed) and quantitatively (direct comparison to experimental culture results). Implemented in BioDynaMo^27^, this is the first ABM known to accurately capture the physical phenomena of biofilms growing under constant fluid flow with experimental validation.

## Results

In this study, the height, detachment rate and event frequency distribution of biofilms produced by the model are compared both qualitatively and quantitatively with data from experimental biofilms obtained from the literature. Each simulation starts with a collection of bacteria and EPS particles spread across the surface from which the mature biofilm emerges. Further analysis for the detachment rate can be found in Supplementary Note 7. Each simulation was repeated 3 times to obtain an average and standard deviation. The results are presented in two forms. First, a collection of simulations with changing EPS production and flow speed. Second, a direct comparison between the model with and without quorum-sensing to an experiment performed by Wilson et al.^28^. Throughout this section, events refer to a clump of bacteria which detaches from the biofilm whose size is determined by the number of bacteria the clump contains.

### Initial simulations

**Fig. 1:**
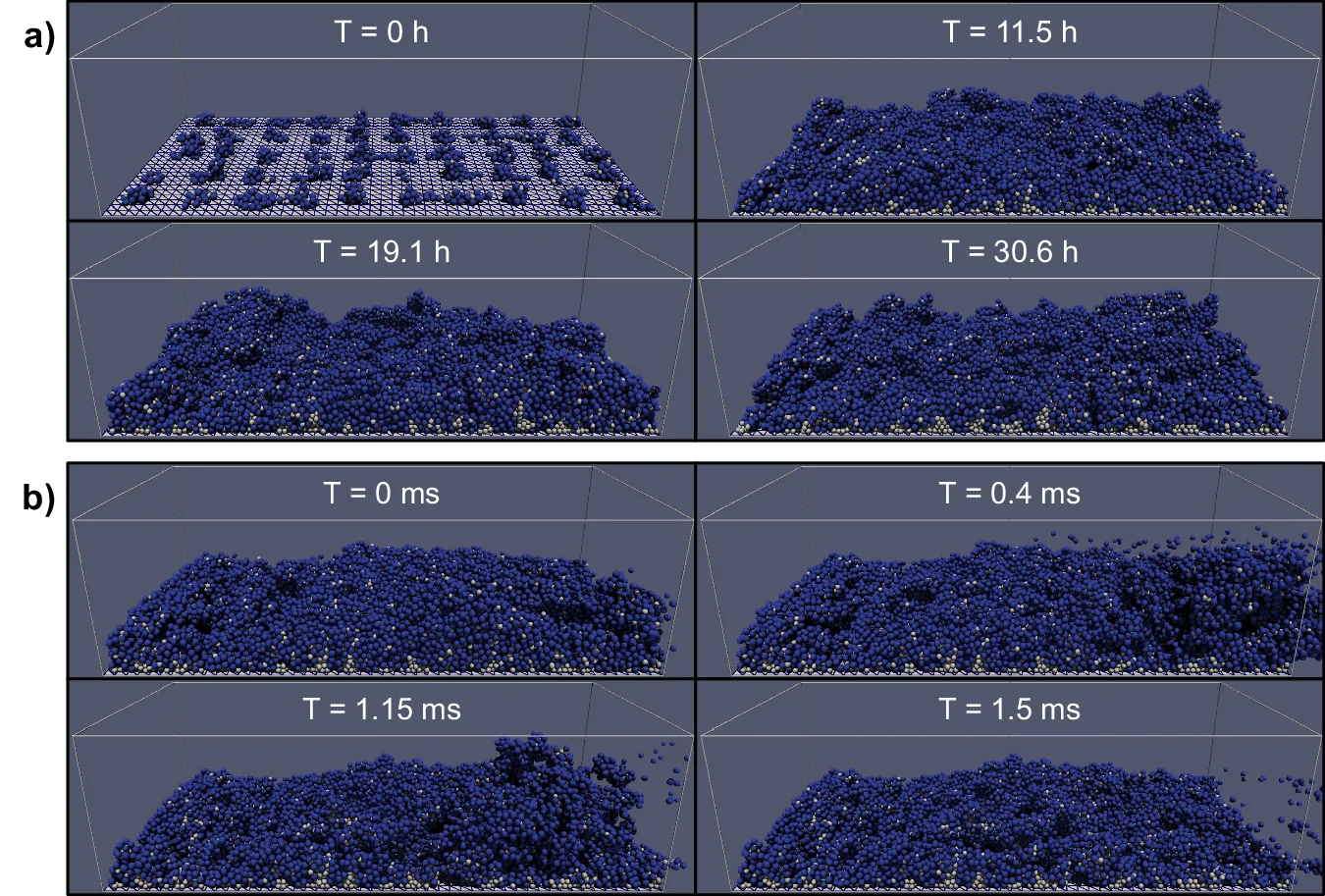
Snapshots of biofilm growth simulation and sloughing event. **a** Snapshots of biofilm growth at 0, 11.5, 19.1 and 30.6 h with 30% EPS production growing under 1.86 mm/s. Blue spheres represent bacteria and white spheres represent particles of EPS. **b** Snapshots of large detachment events at 0, 0.4, 1.15 and 1.5 milliseconds which occurred at timestep 150 (22.9 h).

Figure 1a shows snapshots of the biofilm growing over the course of 1.27 days, with 30% EPS production and a flow speed of 1.86 mm/s. The full simulation can also be viewed in the Supplementary Movie 1. In the beginning of the simulation, only small events consisting of less than 10 bacteria occurred. The rate of detachment is lower than the rate of growth, so the biofilm continues to grow in height. As this biofilm grows, it develops a relatively smooth surface. At around timestep 33, large events occur, reducing the size and increasing the roughness of the biofilm. An example of these large events can be seen in Fig. 1b and Supplementary Movie 2. After this, the biofilm grows in height again until another large event occurs. This cycle of growth and large events keeps the biofilm in a quasi-steady state. The size of the large events and the area of the biofilm they occur in are stochastic; however, their frequency is relatively constant.

**Fig. 2:**
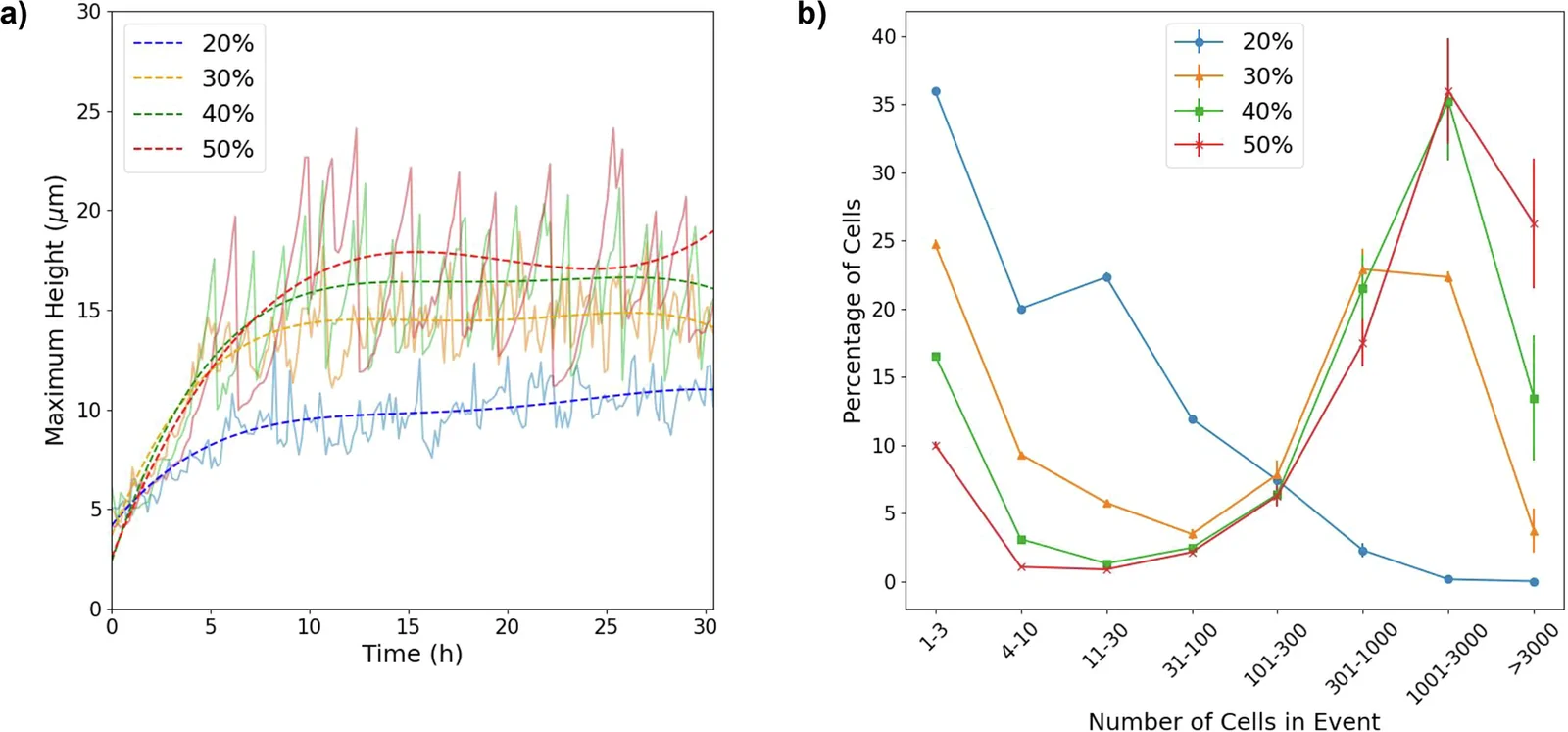
Comparison of biofilm simulations with 20%, 30%, 40% and 50% EPS production in a flow speed of 1.86 mm/s. **a** Biofilm maximum height against time. Dotted lines show polynomial fit of the maximum height that is averaged across three simulations and solid lines show maximum height of one of the repeated simulations as an example. **b** Event frequency distribution expressed as percentage of total cells. Error bars show standard deviation across 3 repeats.

Figure 2a shows the maximum height of this biofilm over the course of the simulation along with three other biofilms operating with an EPS production of 20%, 40% and 50%. To illustrate the changes in the biofilm caused by EPS production, the full growth simulation for the 20% and 50% EPS production can be seen in the Supplementary Movie 3 and Supplementary Movie 4, respectively. The sudden dips in height are caused by large events. As the EPS production increases, the maximum height the biofilm reaches before large events occur increases. The large events also become less periodic but more destructive: removing larger portions of the biofilm in a single time step. The biofilm with 30% EPS production produced the highest average rate of bacteria detachment at 3.71 ± 0.04 cells × 10^4^ min^−1^ mm^−2^, with the 20%, 40% and 50% being 2.59 ± 0.03 cells × 10^4^ min^−1^ mm^−2^, 3.11 ± 0.07 cells × 10^4^ min^−1^ mm^−2^ and 2.30 ± 0.04 cells × 10^4^ min^−1^ mm^−2^, respectively. Figure 2b shows the event frequency distribution expressed as percentages of total detached cells across the entire simulation. Events were grouped into bin ranges based on a logarithmic scale to appropriately quantify the event sizes. At 50% EPS production, the quantity of bacteria detaching via large events is greater than bacteria detaching via small events. As the EPS production lowers, the quantity of bacteria detaching via small and large events become comparable. The 30%, 40% and 50% EPS production simulations show a dip in the frequency of events containing 11–100 bacteria: the vast majority of events are either very small or very large.

**Fig. 3:**
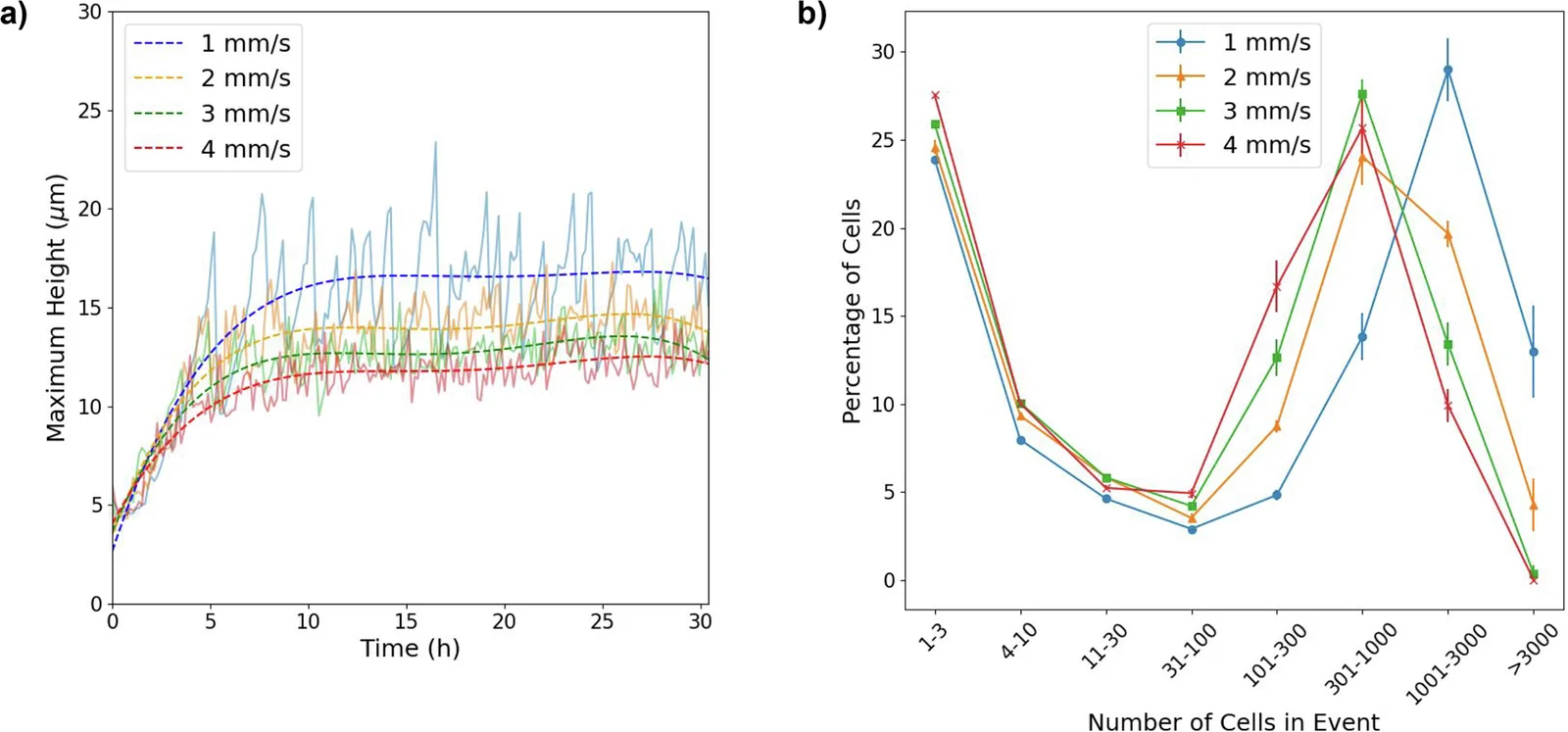
Comparison of biofilm simulations with 30% EPS production in a flow speed of 1 mm/s, 2 mm/s, 3 mm/s and 4 mm/s. **a** Biofilm maximum height against time. Dotted lines show polynomial fit of the maximum height that is averaged across three simulations and solid lines show maximum height of one of the repeated simulations as an example. **b** Event frequency distribution expressed as percentage of total cells. Error bars show standard deviation across 3 repeats.

The flow speed was varied to observe the effect on biofilm growth and detachment. The EPS production was fixed to 30% as it was abundant in both small and large events which allows the influence of flow speed to be the most visible. Figure 3a shows the maximum height of the biofilm growing in a fluid speed of 1 mm/s, 2 mm/s, 3 mm/s and 4 mm/s. As the flow speed increases, the maximum height the biofilm reaches before large events occur decreases. The average rate of detached bacteria steadily decreased with flow speed, with the biofilm growing under 1 mm/s, 2 mm/s, 3 mm/s and 4 mm/s flow speed producing 4.24 ± 0.04 cells × 10^4^ min^−1^ mm^−2^, 3.63 ± 0.04 cells × 10^4^ min^−1^ mm^−2^, 3.26 ± 0.03 cells × 10^4^ min^−1^ mm^−2^ and 2.99 ± 0.03 cells × 10^4^ min^−1^ mm^−2^, respectively. Figure 3b shows the event frequency distribution for each flow speed. The flow speed has a marginal influence on the frequency of small events. The size of the most frequent large event steadily decreases as the flow speed increases.

### Experimental comparison

The experiment chosen to validate the model was performed by Wilson et al.^28^ as it has the most abundant data for comparing event distributions. The experimental setup consisted of a 3 × 3 × 200 mm flow cell connected to a 334 mm long silicon tube which led to a waste container. Inoculation cultures of the nonmucoid clinical isolate PAO1 strain of *P. aeruginosa* were initially grown in a shaker with Lysogeny Broth (LB) (20g/Liter) for 16 h. A 2 mL inoculum was then placed on the flow cell surface and the bacteria were allowed to attach for 45 min. After attachment, the flow is switched on and the biofilm is left to grow for 7 days in 0.02xLB (400 mg/liter) at a flow rate of 1 mL/min in room temperature (22 ^*o*^C).

During the experiment, the biofilm height, detachment rate and event frequency distributions were analysed^28^. The biofilm heights were determined via digital imaging of daily experiments, where stage movement techniques were used to determine the vertical distance between the biofilm-liquid interface and the biofilm-glass interface. The measurements of experimental rate of bacterial detachment considered two aspects- the amount of cells that were leaving in a given event, and the number of times an event of a particular size was occurring. Given the non-normal, skewed size distribution of the results, a Pareto probability distribution was applied to calculate the number of events of specific sizes per mm of surface area per minute. Detached clumps (events) were collected from the effluent daily by taking 1 ml samples of a 1:100 dilution of the effluent. The samples were treated with LIVE/DEAD stain for cell viability evaluation, and flattened onto a filter membrane for confocal imaging, where the area of each flattened clump was used to calculate the total number of cells within a clump. These clumps were then averaged to give an event size frequency distribution.

Given the dimensions of the flow cell used in the experiment, the flow rate is equivalent to 1.86 mm/s. The mixture had a high concentration of LB and was aerated before entering the flow cell, so it is assumed that all bacteria were growing at the maximum specific growth rate throughout the simulation. As the experiment observed roughly equal amounts of erosion and sloughing, the 30% EPS production was selected for the comparison as it provided a similar balance. Two simulations were compared to the experiment: one where bacteria possess the ability to secrete rhamnolipids which are mediated through quorum-sensing (QS+), and one where the bacteria have no quorum-sensing capabilities (QS-). Both simulations were run for 200 time steps equivalent to 1.27 days, allowing the biofilm to operate at its quasi-steady state for one day.

**Fig. 4:**
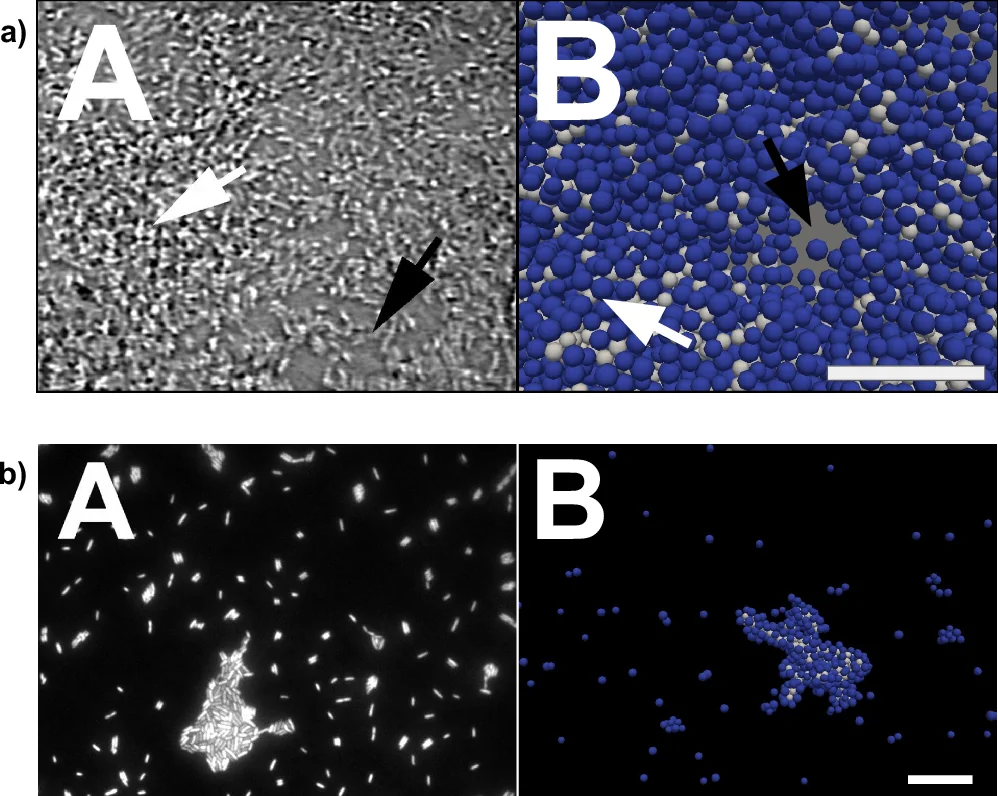
Image comparison between experimental and simulated biofilms and detached biomass. **a** Comparison between experimental (A) and simulated (B) biofilm attached to the surface of the flow cell. Experimental biofilm taken from Figure 3 in Wilson et al.^28^. White arrows point to biofilm mounds and dark arrows point to exposed surfaces. Blue spheres represent bacteria and white spheres represent particles of EPS. Scale bar is set to 10 μm. **b** Comparison between experimental (A) and simulated (B) detached bacteria and biofilm clumps. Experimental biofilm taken from Fig. 4 in Wilson et al.^28^.

Both simulations formed almost identical structures: mounds of bacteria and EPS surrounded by areas of single-layer sheets of bacteria and exposed surface. This structure closely resembles the biofilm observed in the experiment, which are compared in Fig. 4. The average maximum height that both simulated biofilms reached was 15 μm, fluctuating between a minimum of 11 μm and a maximum of 18 μm, resembling the 30% EPS production in Fig. 2a. This is within the range of the biofilm height measured in the experiment; reported as an average between 10 and 22 μm (extracted from Figure 2b in Wilson et al.^28^). The QS+ biofilm gave a slightly higher bacteria detachment rate of 3.82 ± 0.06 cells × 10^4^ min^−1^ mm^−2^ compared to the QS- biofilm of 3.71 ± 0.04 cells × 10^4^ min^−1^ mm^−2^. These are higher than the reported detachment rate in the experiment of 3.02 ± cells × 10^4^ min^−1^ mm^−2^, however the simulation result does fall within the error of the experiment that reported an error of around 90% of the measured detachment rate (extracted from Fig. 2C in Wilson et al.^28^).

**Fig. 5:**
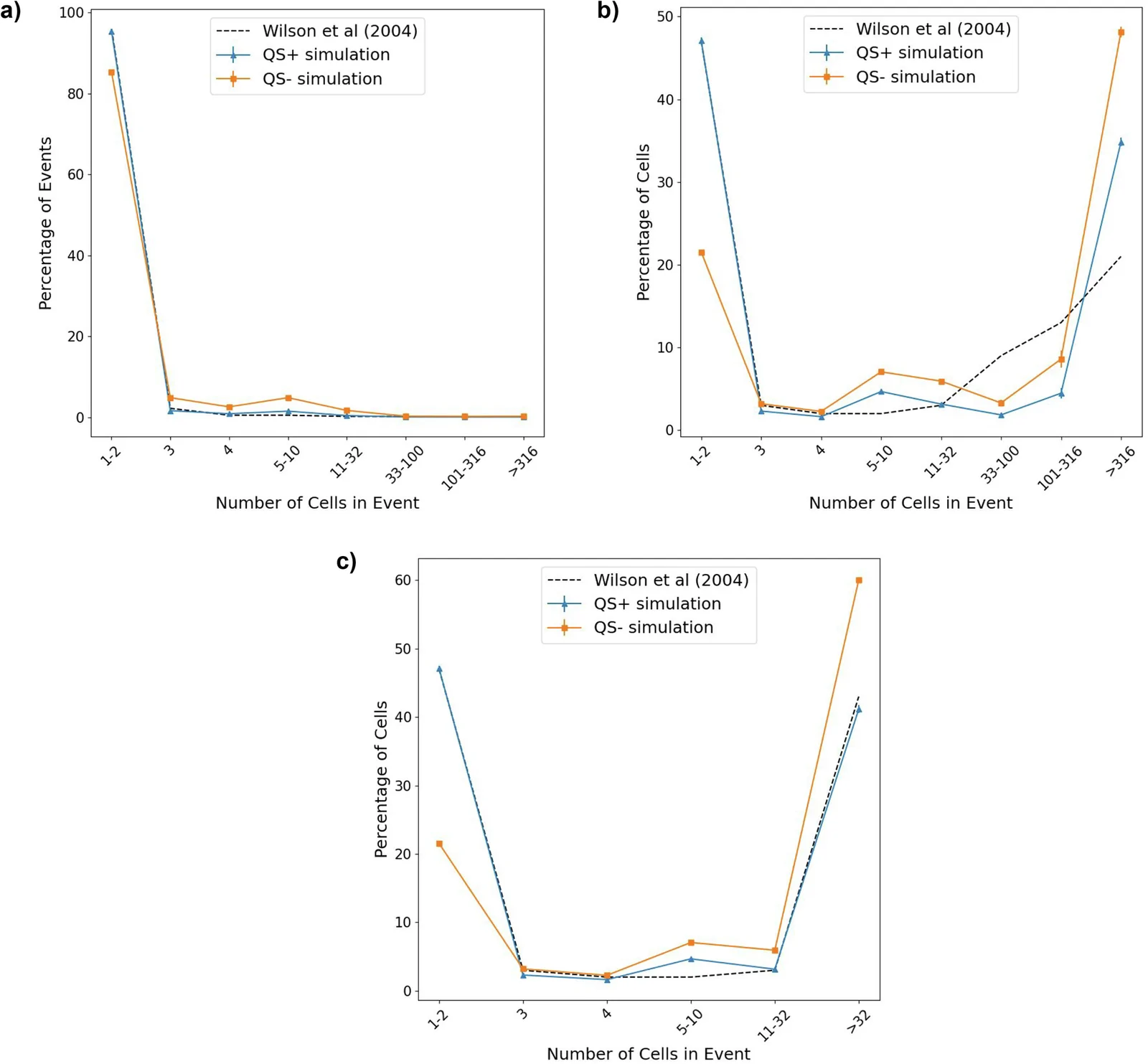
Comparison of event frequency distribution between the QS+ and QS- biofilm simulations and experimental data. **a** Distribution expressed as percentage of total events. Error bars show standard deviation across 3 repeats. **b** Distribution expressed as percentage of total cells with standard bin range. **c** Distribution expressed as percentage of total cells with reduced upper bin range.

The most significant difference between the two simulations is in the event frequency distribution. Figure 5a shows the event frequency distribution expressed as a percentage of total events across the simulation for the QS+ and QS- biofilms and the experiment (extracted from Fig. 6 in Wilson et al.^28^). The bin ranges differ from Figs. 2b and 3b to appropriately compare to the experimental data. Both simulations show strong agreement with the experimental distribution of events, with the QS+ biofilm providing a closer fit. When comparing the event frequency distribution expressed as cells as shown in Fig. 5b, the QS+ biofilm provides a very close fit to the experimental distribution of small events compared to the QS- biofilm. However, both simulations deviate away from the experiment when representing large event sizes. Figure 5c reduces the upper bin range to group the largest events together, which provides a strong fit to the experimental distribution of large events for the QS+ biofilm.

**Fig. 6:**
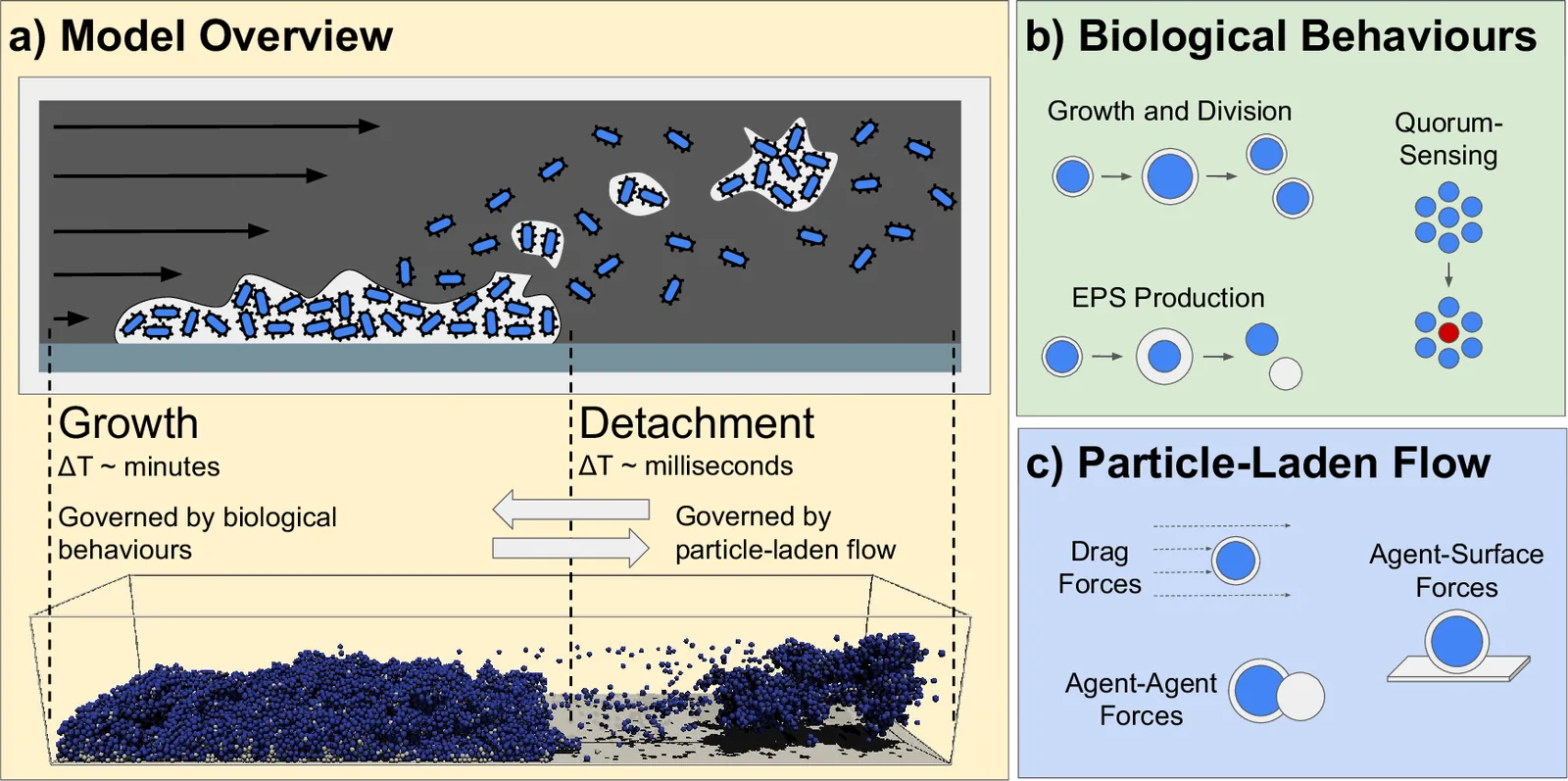
Schematic of model overview. **a** The simulation cycles between growth and detachment each timestep. **b** Biological behaviours which govern growth. **c** Physical forces which govern particle-laden flow.

## Discussion

*P. aeruginosa* biofilms growing in flow often reach a quasi-steady state between growth and detachment^28–31^, replicated in our simulations. Differences are seen in the average height at steady state and the frequency of growth/sloughing cycles. Sloughing occurs when shear force overcomes the biofilm’s cohesive strength. Biofilm height indicates potential sloughing because flow speed increases with distance from the surface. Increasing EPS production strengthens cohesion, leading to a higher biofilm^32^. Faster flow speed increases shear force closer to the surface, causing sloughing at lower heights^33^. The frequency of growth and sloughing cycles is largely dependent on the size of sloughing events. Increasing the EPS production in the simulated biofilm leads to the biofilm becoming more bound to itself than the surface it grows on^34^. This results in more destructive sloughing events, requiring longer regrowth time^29^. Thinner biofilms (low EPS, fast flow) prevent large clumps, blurring the line between sloughing and erosion^35^.

The simulated biofilm initially begins as a dense flat block of bacteria and EPS, but over time becomes rough; forming mushroom-like structures surrounded by a bacterial monolayer commonly reported from in vitro experiments of *P. aeruginosa* biofilms growing in flow^8,36,37^. These mushroom-like structures appear soon after the first sloughing events due to the spherical shape of these structures being the most resistant to shear stress. The 20% EPS production simulation and biofilms simulated under faster flow speeds formed flat biofilms, most likely due to lack of height^33^. Interestingly, the 50% EPS production simulation had less pronounced mushroom shapes compared to the 30% and 40% EPS production simulations. As the sloughing events were more destructive, the remaining biofilm had a similar distribution of bacteria across the surface as the beginning of the simulation, leading to the same flat biofilm^38^. This implies that for the mushroom shapes to appear, sloughing events need to be smaller to create an uneven distribution of bacteria across the surface. Introducing quorum-sensing had no effect on the simulated biofilm shape and size, which was also found by Davey et al.^39^ when observing biofilm growth of rhamnolipid-deficient strains of *P. aeruginosa*.

The detachment rate of bacteria predictably decreased with smaller simulated biofilms^33^, which is most evident from the flow speed analysis. The most interesting deviation from this trend is the peak in detachment rate for 30% EPS production despite producing smaller biofilms compared to 40% and 50% EPS production. Larger EPS production prevents bacteria from escaping the biofilm so the majority of bacteria are released in short bursts via sloughing, which become less frequent with increasing EPS production. This suggests that for the biofilm to become the best ‘factory’ for releasing bacteria into the surrounding fluid there needs to be a balance of bacteria detaching via erosion and sloughing. The dip in detachment frequency for clumps containing 11–100 bacteria in almost all simulations has also been reported from previous experiments^28,40^. This may be due to the web-like structure of the EPS matrix^41^: a bacterium will detach from the biofilm because it has either failed to adhere to the EPS shortly after division or the EPS matrix as a whole detaches. The increase in detachment rate for the simulated biofilm capable of producing rhamnolipids has been reported in previous experiments^42^. It is worth noting that this increase was only around 3% whereas previous experiments have observed 100% to 800% increase when comparing rhamnolipid-deficient strains to wild type strains of *P. aeruginosa*^42^. This could be due to the simulated biofilm being smaller than typical in vitro experiments, as the number of bacteria which switch phenotypes to produce rhamnolipids exponentially increases with biofilm thickness.

The simulation was able to capture the shape and height of the biofilm grown in the experiment by Wilson et al.^28^. It is worth noting the published experiment reported a height of 30 μm by the end of the 7 days so the simulated biofilm is more reflective of the first three days of the experiment. The simulation also captured the detachment rate of the published experiment, however, this was only achieved by increasing the growth rate of the bacteria to match the doubling times typically reported of the PA01 strain in LB broth^43^ compared to the doubling time reported by Wilson et al.^28^. Furthermore, the simulation provided good agreement with the experimental detachment event frequency distribution once the quorum-sensing behavior was included for the bacteria.

The most significant discrepancy between the simulation and the published experiment can be seen in the size of sloughed clumps: the simulation overrepresented the largest clumps and underrepresented smaller clumps consisting of 33-316 bacteria. The model measures clump sizes within 2 milliseconds of detachment from the biofilm, whereas the experiment measured clump sizes after they had traveled across the flow cell and through the connecting tube that led to the effluent collection. This longer traveling time would give large clumps the opportunity to break up when colliding with other clumps or with the walls of the flow cell/tube, which may account for the difference^15^. These simulations and previous experiments have noted that sloughing is a common method for bacteria at the center of the biofilm to be released into the surrounding fluid^44^. As evidence has suggested that the most infectious bacterial variants reside at the center of the biofilm^45^, this could explain why some experiments have reported that clumps detaching from biofilms could be more infectious than large collections of individual cells^46^, highlighting the importance of the models ability to simulate sloughing. Another noteworthy discrepancy is that our model assumes the bacteria to be spherical, whereas *P. aeruginosa* are rod-shaped^6^. While one might assume the difference in collision physics between rods and spheres may influence detachment, the agreement between the simulation and the experiment of Wilson et al. implies that modelling spheres may be an adequate approximation of rods.

There is growing evidence that species-specific biological behaviours play a significant role in biofilm formation and bacterial detachment^47^. As this model contains an experimentally-validated description of the physical mechanisms influencing biofilms, discrepancies found between specific experiments and common observations of biofilms in fluid flow discussed earlier are indirectly attributed to biological mechanisms excluded from the model. Henzter et al.^48^ and Stoodley et al.^40^ have reported flat biofilms produced by *P. aeruginosa* growing under laminar flow compared to the commonly-accepted mound shapes, which they attributed to using a certain growth medium. Evidence suggests that different nutrient compositions in the growth medium can influence EPS production rate^49^ which, as shown in this study, can change the biofilms shape in fluid flow. Heydern et al.^50^ discovered that motility and quorum-sensing led to flat biofilms by evenly spacing bacteria across the surface^51^. The flat biofilms formed from the 20% and 50% EPS production simulations were caused by evenly-spaced bacteria on the surface after a sloughing event. In this study, the simulation matched the experimental detachment event frequency distribution once the quorum-sensing capabilities were included, further illustrating the point that many differences found between simulations and experimental results can be resolved by including specific biological behaviours in the model.

To the authors’ knowledge, the model presented here is the first ABM of biofilms to capture the physical phenomena of biofilms growing under constant fluid flow. The simulations produced by the model accurately captured erosion and sloughing as natural byproducts of bacterial growth and EPS production. Experimental parameters used as input to the model can reflect the behavior of lab-grown biofilms. While simulations in this study focused on *P. aeruginosa* biofilms, future ABMs could expand on this model by including additional biological and chemical rules such as chemotaxis and nutrient diffusion and advection to simulate other biofilm-forming species or replicate specific experiments with minimal addition to computational expense.

The model’s most significant shortcoming is computational expense. Particle-laden flow simulations are notorious for being computationally demanding. Improving collision physics to allow for stable simulations with larger time steps would decrease the simulation run time. The simulation results can also be sensitive to the many parameters the model takes as input; most importantly the physical parameters such as contact and attraction force strength which tends to be experimentally ill-defined. Determining these physical parameters experimentally can improve the accuracy of the model and its reliability in predicting future experiments. It is worth noting that while the model was capable of replicating the experiment by Wilson et al using a spherical approximation for bacteria shape, this may differ for different experiments as evidence suggests that bacteria shape do play a role in biofilm formation^52^.

Researching optimal designs for medical equipment and hospital plumbing which minimise the spread of *P. aeruginosa* and other nosocomial infections are costly endeavours, mainly due to the trial-and-error associated with finding the best result. To cut down on experimental time, researchers can use this model to predict how environmental factors such as flow speed, density and viscosity can influence biofilm shape and detachment and sloughing rate to design future experiments accordingly. Similarly, biological parameters such as growth speed and EPS production rate can also be used to predict how biofilm survivability can change when produced by different bacterial strains. By using this model to make predictions on future experiments, biofilm researchers could bring down laboratory time and costs.

## Methods

The model functionally operates as a collection of particle-laden flow simulations^53^ strung together by periods of growth, division, EPS production and quorum-sensing. A schematic of the model is given in Figure 6. The biofilm resides in a 100 × 50 × 50 μm box which represents a small slice of the whole biofilm assumed to represent the average behaviour. The surface is initially covered by collections of bacteria and EPS which then grow into a mature biofilm. Each timestep is broken down into two parts: growth, division, EPS production and quorum-sensing in the order of minutes (Fig. 6b) followed by the particle-laden flow model in the order of microseconds (Fig. 6c). The coupling between these two timescales relies on the steady state reached at the end of the particle-laden flow, where it is assumed the biofilm will not change shape until significant growth of the biofilm occurs. At the end of each timestep, the height of the biofilm, number of detached bacteria and detached clump sizes are recorded.

This model is created using the BioDynaMo software^27^, whose highly parallelised and customisable framework minimises computational expense for complex ABMs. Growth, division, EPS production and quorum-sensing are added as behaviours assigned to agents, which are automatically distributed equally to each thread throughout the simulation. The agent collision forces utilise BioDynaMo’s nearest-neighbour search function to minimise the time of the most computationally expensive aspect of the particle-laden flow. BioDynaMo operates on a scheduler, which allows for full customisation of the order of operations throughout a given timestep.

The agents are defined as spherical and incompressible. The model contains two types of agents: *P. aeruginosa* cells and particles of EPS. Both types are capable of repulsion and attraction to each other and the surface, while only the cells can grow, divide, produce EPS, and utilise quorum-sensing. The following sections will discuss how the behaviors of *P. aeruginosa* are modeled and provide details of the particle-laden flow model, as well as the simulation algorithm and post-simulation analysis. Throughout these sections, *P. aeruginosa* are referred to as cells, particles of EPS are referred to as EPS, and agents refer to both. All values used for the parameters presented in this section can be found in Supplementary Note 1.

### Biological behaviors

Cells will consume the nutrients in their immediate surroundings to grow, divide and manufacture EPS. This model uses the monod equation to simulate bacterial growth, commonly used by other biofilm models^22,54^:

1 $$\frac{d{m}_{cell}}{dt}=\mu (1-f){m}_{cell},$$

where *m*_*cell*_ is the mass of the bacteria, *μ* is the growth rate and *f* is the fraction of biomass dedicated to producing EPS^55^ which throughout this study is referred to as the EPS production. In this work, it is assumed that the environment is saturated with nutrients so all cells grow at the same rate. The cell will grow in size until its diameter reaches a threshold and divides, producing a daughter cell and donating a uniformly random portion of its mass to the daughter between 45% - 55%^20^.

The approach to modeling EPS production follows the particle approach of previous models^19,22^. A cell will accumulate EPS as a shell around itself, whose size is determined by:

2 $$\frac{d{m}_{EPS}}{dt}=\mu f{m}_{cell},$$

where *m*_*EPS*_ is the mass of the EPS shell. Once the ratio between the shell diameter and cell diameter reaches a threshold, the entire shell will peel away from the cell and become its own agent.

To capture dispersion in the model, cells are given a quorum-sensing behaviour modeled using the neighbour-searching algorithm in BioDynaMo^27^. After growth, division and EPS production, each cell will search for all other cells in a given radius. If the number of cells within the search radius is above a threshold, the cell will switch phenotype. It is assumed once the cells have switched phenotype, they will produce rhamnolipids and swim out of the biofilm^56^, thus to save computational expense these cells are removed from the simulation at the beginning of the particle-laden flow. Further information about the quorum-sensing model can be found in Supplementary Note 2.

### Particle-laden flow model

Once the cells have completed their biological behaviours, the increase in agent population causes a rise in pressure inside the biofilm due to overlapping agents. To relieve the pressure, as well as simulate erosion and sloughing of cells and EPS, a particle-laden flow model is implemented to calculate the change in biofilm shape and size. The model implemented is commonly used for engineering applications^57^ but has been used in previous biofilm models^19,21^. The forces that all agents experience can be split into three parts; agent-agent forces, agent-surface forces and the drag force.

The force between an agent *i* and a neighbouring agent *j* consists of a contact force and attraction force. The contact force is modeled using the spring-dashpot model^58^:

3 $$\vec{{F}_{ij}^{ac}}={k}^{n}\delta \widehat{{n}_{ij}}-{\gamma }_{ij}^{n}{m}_{ij}\vec{{v}_{ij}},$$

where *k*^*n*^ is the normal spring constant, $\delta \widehat{{n}_{ij}}$ is the overlap displacement between the agent surfaces, ${\gamma }_{ij}^{n}$ is the normal damping constant, *m*_*ij*_ and $\vec{{v}_{ij}}$ is the effective mass and relative normal velocity between the two agents, respectively. The attraction is modeled using the Van der Waals force between two spheres^59^, defined as:

4 $$\vec{{F}_{ij}^{aa}}=-\frac{{H}^{a}{R}_{e}}{6{\delta }^{2}\widehat{{n}_{ij}}},$$

where *H*^*a*^ and *R*_*e*_ is the Hamaker constant and effective radius between the two agents, respectively. To prevent singularities when agent surfaces make contact, a restriction is applied to the overlap distance such that it cannot fall below a minimum separation distance *h*_*min*_^60^. It is appreciated that the EPS acts as the binding agent which holds the biofilm together^61^, thus this model assumes the attraction force for EPS particles is three orders of magnitude higher compared to cells, matching previous models^21^.

An agent experiences contact from five out of the six boundaries *k* of the domain, the only excluded boundary being the maximum *x* boundary which is considered to be open. The contact force is modeled using a modified version of the spring-dashpot model:

5 $$\vec{{F}_{ik}^{bc}}={k}^{n}\delta \widehat{{n}_{ik}}-{\gamma }_{i}^{n}{m}_{i}\vec{{v}_{ik}^{n}},$$

where $\delta \widehat{{n}_{ik}}$ is the overlap distance between the agent surface and the boundary, *m*_*i*_ is the mass of the agent and $\vec{{v}_{ik}^{n}}$ is the normal velocity between the agent and the boundary. An agent will also experience a friction and attraction force to the surface located at the minimum *z* boundary. The friction force is also modeled using the modified spring-dashpot model:

6 $$\vec{{F}_{i}^{sf}}={k}^{t}\vec{{U}^{t}}-{\gamma }_{i}^{t}{m}_{i}\vec{{v}_{i}^{t}},$$

where *k*^*t*^ is the tangential spring constant, $\vec{{U}^{t}}$ is the tangential displacement, ${\gamma }_{i}^{t}$ is the tangential damping constant and $\vec{{v}_{i}^{t}}$ is the tangential velocity between the agent and the surface. Details of the calculation of the normal and tangential damping constants can be found in Supplementary Note 3. The attraction force is modeled using the Van der Waals force between a sphere and an infinite plane^59^:

7 $$\vec{{F}_{i}^{sa}}=-\frac{{H}^{s}{R}_{i}}{6{\delta }^{2}\widehat{{n}_{i}}},$$

where *H*^*s*^ is the Hamaker constant between the agent and surface, *R*_*i*_ is the radius of the agent and ${\delta }^{2}\widehat{{n}_{i}}$ is the overlap displacement between the agent surface and the surface. The same restriction on the overlap distance that was applied to the agent-agent attraction force is also applied to the agent-surface attraction force.

The drag force is modeled using the Syamlal-O’brien drag model for particle beds^62^, which is defined as:

8 $$\vec{{F}_{i}^{d}}=\frac{\pi }{8}\frac{{d}_{p}^{2}{\rho }_{f}}{{V}_{r}^{2}}{C}_{d}| \vec{{U}_{if}}| \vec{{U}_{if}},$$

where *d*_*p*_ is the diameter of the agent, *ρ*_*f*_ is the density of the fluid, *V*_*r*_ is the relative velocity correlation, *C*_*d*_ is the drag coefficient and $\vec{{U}_{if}}$ is the relative velocity between the agent and the fluid. Further details on the calculation of the latter three variables can be found in Supplementary Note 4 and 5.

All forces listed above are combined together in a Lagrangian framework to give a change in velocity of the agent:

9 $${m}_{i}\frac{d\vec{{v}_{i}}}{dt}={\sum }_{j\ne i}^{N}(\vec{{F}_{ij}^{ac}}+\vec{{F}_{ij}^{aa}})+{\sum }_{k=0}^{5}(\vec{{F}_{ik}^{bc}})+\vec{{F}_{i}^{sf}}+\vec{{F}_{i}^{sa}}+\vec{{F}_{i}^{d}}.$$

BioDynaMo utilizes a uniform grid nearest-neighbour search algorithm to find the nearest agents within a 1.58 μm search radius. All neighbouring agents *N* found within this radius are considered when calculating the net force from agents.

The simulation takes place close to the surface in a niche area of computational fluid dynamics called the laminar sub-layer^63^. This region is too close to the boundary for turbulent flow to occur, thus it is assumed the fluid flow in the region is laminar. The Reynolds number for an agent in the model is on the order of 10^−6^, which is typically reported for bacteria^64^. Stoke’s flow is incorporated in the simulation to calculate the fluid flow, whose momentum equation is^65^:

10 $${\mu }_{f}{\nabla }^{2}\vec{{U}_{f}}=\nabla {p}_{f},$$

where *μ*_*f*_ is the dynamic viscosity, $\vec{{U}_{f}}$ is the fluid velocity and *p*_*f*_ is the fluid pressure. The boundary conditions for the fluid dynamics are as follows: minimum and maximum *x* has open boundaries, minimum and maximum *y* has symmetrical boundaries, maximum *z* boundary has a fixed fluid velocity *U*_*b*_ and minimum *z* boundary has no slip conditions. Before the simulation begins, the fluid velocity field is calculated in OpenFoam^66^; an open-source complex fluid dynamics solver. The field values are then mapped to each voxel in the uniform grid in BioDynaMo. The model excludes particle influence on the fluid to reduce computational expense.

### Model algorithm

Before the simulation begins, the fluid velocity field is calculated in OpenFoam and transferred to BioDynaMo. Bacteria and EPS positions are initialised and the simulation loop begins. The simulation loop is split into three parts: biological behaviours, relaxation and particle-laden flow. The bacteria grow, divide, produce EPS, and switch phenotype. This raises the pressure in the biofilm and creates extreme overlap between agents. To separate these agents, the maximum velocity of the agents are restricted to 1 mm/s to allow the agents to separate until the system is stable enough to begin the particle-laden flow. The system is considered relaxed once the net force for all agents is less than 0.5 nN. All bacteria that switched phenotype are recorded for post-simulation analysis and removed from the simulation. The velocity restrictions on the agents are removed and the particle-laden flow begins. Agents which pass the maximum *x* boundary continue traveling until the steady state is reached. The steady state for the particle-laden flow is found when either all agents within the bounds of the system have moved less than 0.1 μm in 10 microseconds or the particle-laden flow has exceeded 1.5 × 10^5^ iterations. Once the steady state is found, it is assumed the biofilm will remain unchanged until a significant amount of growth, division and EPS production occurs so these behaviours are calculated again. This loop repeats until the specified number of time steps have been reached. All agents that have passed the maximum *x* boundary or are still moving by the end of the steady state calculation are frozen in place and their distribution is recorded for post-simulation analysis and then removed from the simulation. A flow chart for the simulation can be found in Supplementary Note 1.

### Post-simulation analysis

The key difference between erosion and sloughing is the size of the biofilm clumps that detach from the biofilm^13,14^. In this study, these clumps are referred to as events. An event is defined as a collection of detached agents which are in contact with at least one other agent in the event. A single event can consist of a lone bacterium or a large collection of bacteria connected together by strings of EPS particles.

To categorise these events, a distance-threshold clustering algorithm was created to group agents together based on distance between their surfaces. The algorithm starts by retrieving the distribution of detached agents recorded at the end of the particle-laden flow and compiling them into a list. The algorithm will take the first agent in the list and give it a unique event ID and search for all neighbouring agents within a radius of 0.5 μm (sensitivity analysis for this value can be found in Supplementary Note 6). Any neighbouring agent within range is given the same event ID and their position is moved to the top of the list. The original agent is then removed from the list. If the next agent in the list already has an event ID, it will spread that ID to all neighbouring agents and move them up the list. If the next agent does not have an event ID, it is given a new one and searches. This cycle repeats until the agent list is empty, resulting in every agent being given an event ID. These IDs can then be used to identify how many bacteria are in the same event. All bacteria which detach via quorum-sensing are assumed to be individual events. This returns a list of all the events that occurred in a single time step defined by the number of detached bacteria it contains. This algorithm is repeated for all time steps in the simulation to give an event frequency distribution across the simulation.

Along with analysing events, the biofilm is assessed through measuring its maximum height and total number of bacteria which detach over the course of the simulation. The detached bacteria are averaged across all time steps and scaled up to give an average number of detached bacteria per minute per mm^2^.

## Supplementary information

Supplementary information

Supplementary Movie 1

Supplementary Movie 2

Supplementary Movie 3

Supplementary Movie 4

## Data Availability

Simulation data can be replicated by running the simulations generated by the code created for the project. All experimental data were taken from Wilson et al (2004). The code created for the project is available in the BioDynaMo repository list on GitHub: https://github.com/BioDynaMo/Biofilm-Growth-in-Flow.

## Code availability

The code created for the project is available in the BioDynaMo repository list on GitHub: https://github.com/BioDynaMo/Biofilm-Growth-in-Flow.
