# Supplementary material for: Agent-based modeling of erosion and sloughing during growth of Pseudomonas aeruginosa biofilms
Source: NPJ Biofilms Microbiomes. 2026 Jun 22;12:204. doi: 10.1038/s41522-026-01052-1 (PMC13669389; doi:10.1038/s41522-026-01052-1)
Supplement: Supplementary file 1 [file 41522_2026_1052_MOESM1_ESM.pdf]

Supplementary Information

Ryan Bournes, Suzanne Hingley-Wilson, Bing Guo,
Cayla Harris, Umar Abubacar, Mark Chambers, Roman Bauer

1 Parameter List and Flow Chart

Supplementary table 1 displays the list of parameters used in all simulations for this study. The parameters were taken from either measured parameters for *Pseudomonas aeruginosa*, previous ABMs of biofilms or chosen to produce an accurate simulation. All parameters are listed in SI units. Supplementary Figure 1 presents a flow chart of the model.

2 Quorum Sensing Parameters

Quorum-sensing (QS) involves the production of autoinducers by bacteria which diffuse through the biofilm and bind to the receptors of neighbouring bacteria. Once a bacterium detects a concentration of autoinducers above a certain threshold, it switches phenotype. Modeling QS by the diffusion of autoinducers requires four parameters: an autoinducer production rate, diffusion coefficient, decay rate and threshold for phenotype switching. Unfortunately, these parameters can be difficult to measure as all four of these parameters can vary according to bacterial species and strain, type of autoinducer, type of receptor, phenotype associated with QS, nutrient concentration, growth medium, temperature, pH and cell cycle phase. As these parameters are still unknown for triggering rhamnolipid production in *P. aeruginosa*, the QS parameters in this model were fitted to closely match the data presented in Wilson et al [5]. Thus, a model for QS with only two parameters (search radius and threshold) was chosen to make this process simpler.

The QS search radius and threshold have a cubic relationship. For example, multiplying the search radius by 2 and multiplying the threshold by 8 will keep the dispersion mostly unchanged. However a smaller search radius and threshold causes the dispersion to become more localised in population-dense regions of the biofilm rather than spread across the entire simulated biofilm. Thus to observe the potential influence of dispersion on the biofilm morphology, particularly for a biofilm which represents the experiment on a smaller scale, the search radius is kept short to 3 μm.

To determine the value for the QS threshold, a range of values were tested simply through trial-and-error. Supplementary figure 2 provides a sensitivity analysis for this parameter when comparing to the experimental results of event

Parameter	Symbol	Value	Unit	Source
**Biological Parameters**				
Diameter of cell	d	10^{-6}	m	[1]*
Growth Rate	μ	3.3×10^{-4}	s^{-1}	[2]†
Fraction of biomass converted to EPS	f	$0.2 - 0.5$	kg/kg	Chosen
Diameter for division	d_{div}	1.3×10^{-6}	m	[3]
Diameter for EPS particle excretion	d_{EPS}	1.2	m/m	[3]
Quorum-sensing search radius	r_{QS}	3×10^{-6}	m	Chosen‡
Quorum-sensing threshold	N_{QS}	52	N/A	Chosen‡
**Particle Parameters**				
Particle density	ρ_p	1000	kg/m^3	[4]
Normal spring constant	k^n	0.1	kg/s^2	Chosen
Tangential spring constant	k^t	0.01	kg/s^2	Chosen
Coefficient of restitution	e	10^{-30}	ms/ms	Chosen
Agent-agent Hamaker constant	H^a	1.6×10^{-21}	$kg\ m^2/s^2$	[4]
Agent-surface Hamaker constant	H^s	2.3×10^{-21}	$kg\ m^2/s^2$	[4]
Minimum separation distance	h_{min}	5×10^{-9}	m	Chosen
**Fluid Parameters**				
Fluid velocity in bulk	U_b	$0.001 - 0.004$	m/s	Chosen
Fluid dynamic viscosity	μ_f	0.001	kg/ms	Water at 21 ^{o}C
Fluid density	ρ_f	1000	kg/m^3	Water at 21 ^{o}C
**Resolution Parameters**				
Fluid domain resolution	N_f	$50 \times 25 \times 25$	N/A	Chosen
Biological timestep length	Δt_{bio}	550	s	Chosen
Physical timestep length	Δt_{phy}	10^{-8}	s	Chosen

Supplementary Table 1: **List of model parameters used in this study.**
* *P. aeruginosa* are rod-shaped, with a width of 0.5-1 μm and a length of 1-5 μm.
† determined from a doubling time of 35 minutes for *P. aeruginosa* in Lysogeny Broth.
‡ reason given in section 2.

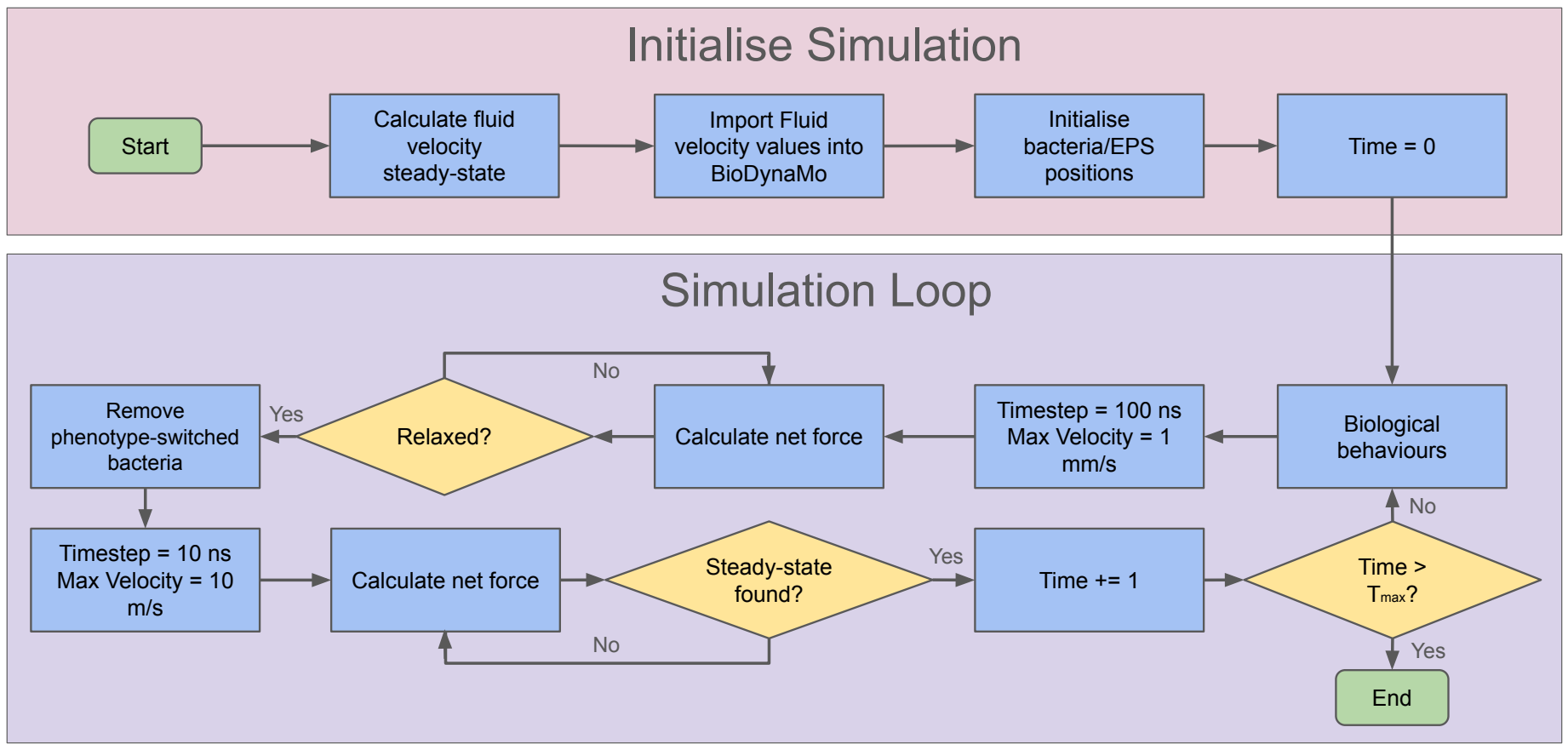

Supplementary Figure 1: **Flow chart of the model algorithm.** The model cycles between biological behaviors, relaxation and particle-laden flow to simulate changes in the biofilm after each period of growth.

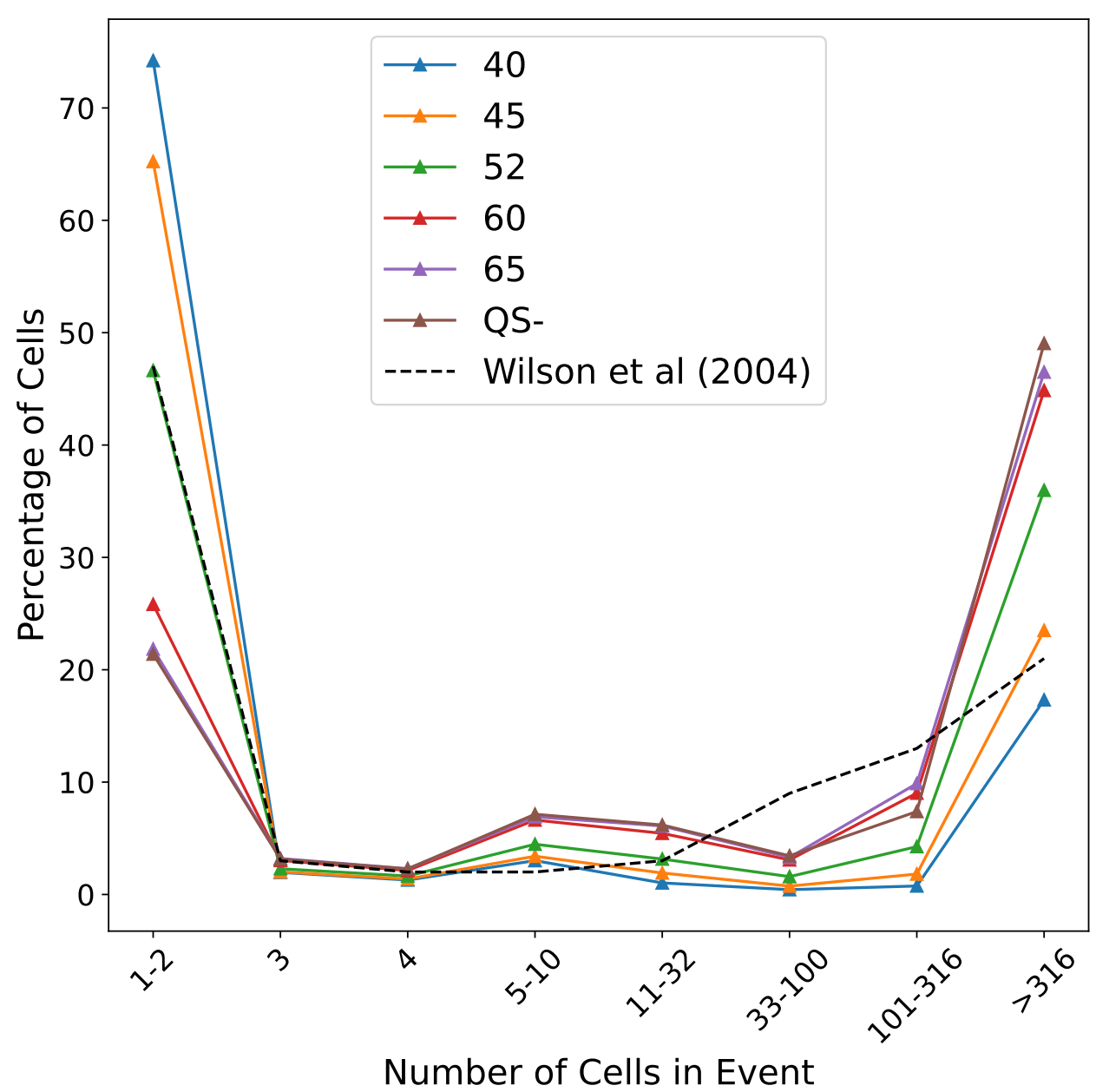

Supplementary Figure 2: **Sensitivity analysis for the bacteria count threshold to trigger phenotypic change.** As threshold increases, the results match the QS- simulation. As threshold decreases, more bacteria switch phenotype and detach as individuals and large biofilm clumps become less populated.

frequency distribution. As the threshold increases, the distribution tends towards the QS- simulation results. This is to be expected as setting the threshold to a high value prevents any bacteria from switching phenotype. Decreasing the threshold leads to more bacteria switching phenotype and thus leaving as individuals. This also leads to less bacteria contained in clumps. While the physical size of the clumps is mostly the same, the proportion of bacteria compared to EPS in the clump significantly reduces when decreasing the QS threshold.

The detachment rate of the simulation peaks near 52 bacteria as the threshold with 3.82 cells $\times$ 10^4 min^{-1} mm^{-2}. Detachment rate plateaus at higher thresholds to match the QS- simulation around 3.71 cells $\times$ 10^4 min^{-1} mm^{-2}. The detachment rate significantly decreases as the threshold decreases with the 45 and 40 bacteria count threshold simulations providing 3.56 cells $\times$ 10^4 min^{-1} mm^{-2} and 3.16 cells $\times$ 10^4 min^{-1} mm^{-2}. Interestingly, the 40 threshold simulation provides a closer fit to the experimental detachment rate compared to the 52 threshold simulation. However, the 52 threshold simulation was chosen as the main comparison to the experimental results as it provided a closer fit to the event frequency distribution compared to the 40 threshold simulation.

3 Damping Constant

The normal and tangential damping constants in the spring-dashpot model are calculated using the following formula:

$$\gamma^n = -2ln(e)\left(\sqrt{\pi^2 + ln(e)^2}\sqrt{\frac{m*}{k^n}}\right)^{-1}, \tag{1}$$

$$m* = \begin{cases} \frac{m_i m_j}{m_i + m_j}, & \text{for agent-agent collisions,} \\ m_i, & \text{for agent-surface collisions,} \end{cases}$$

$$\gamma^t = -1ln(e)\left(\sqrt{\pi^2 + ln(e)^2}\sqrt{\frac{m_i}{k^t}}\right)^{-1}, \tag{2}$$

where m_i and m_j is the mass of the agent i and a neighbouring agent j, respectively.

4 Details on Drag Force Calculation

This section includes further details of the Syamlal-O'brien drag model calculation [6]. The drag coefficient used in the model is calculated using

$$C_d = \left(0.63 + 4.8\sqrt{\frac{V_r}{Re}}\right)^2, \tag{3}$$

where V_r is the relative velocity correlation and Re is the particle Reynolds number defined as

$$Re = \frac{|\vec{U_{if}}|d_p}{\mu_f}, \tag{4}$$

where $\vec{U_{if}}$ is the relative velocity between the agent and the fluid, d_p is the agent diameter and μ_f is the kinematic viscosity of the fluid. The relative velocity correlation increases the strength of the drag force in regions of dense agents and is calculated using:

$$V_r = 0.5(A - 0.06Re + \sqrt{(0.06Re)^2 + 0.12Re(2B - A) + A^2}), \tag{5}$$

$$A = \alpha_f^{4.14}, \qquad B = \begin{cases} \alpha_f^{2.65}, & \alpha_f < 0.15 \\ 0.8\alpha^{1.28}, & \alpha_f \geq 0.15 \end{cases}$$

where α_f is the fluid fraction. The particle fraction α_p is calculated by summing the volumes of each agent whose center lies within a voxel of the fluid field then dividing by the volume of the voxel, which is then converted to the fluid fraction using $\alpha_f = 1 - \alpha_p$. This is then repeated for each voxel to get a fluid fraction field, which is updated every 100 iterations of the particle-laden flow. The particle fraction is prevented from exceeding 0.95 to prevent singularities in the drag force.

5 Relative Velocity Calculation

The contact force is modeled using the spring-dashpot approach. The collision time T_c, which is the time two agents are in contact with one another during a collision, can be calculated from parameter inputs to the spring-dashpot model:

$$T_c = \sqrt{\pi^2 + ln(e)^2}\sqrt{\frac{m_{eff}}{k^n}}, \tag{6}$$

where m_{eff} is the effective mass of the colliding agents. For a given collision in the simulation, the collision time is on the order of 10^{-6} seconds. For a stable simulation, the physical timestep needs to be $\tilde{1}00$ times less than the collision time, leading to a physical timestep of 10^{-8} seconds. For an agent moving at the bulk fluid speed of around 1 mm/s to leave the simulation space of 100 μm, the number of physical time steps to find the physical steady state (or at least empty the simulation of moving agents) would be on the order of 10^7, which is far too computationally expensive. To overcome this, the bulk fluid velocity when calculating the fluid velocity field is set to 1 m/s. The relative velocity between the agent and the fluid $\vec{U_{if}}$ the agent experiences is then calculated as:

$$\vec{U_{if}} = (\vec{U_f^*} - \vec{U_i})\vec{U_f}, \tag{7}$$

where $\vec{U_f^*}$ is the fluid velocity in the simulation, $\vec{U_i}$ is the velocity of the agent and $\vec{U_f}$ is the fluid velocity in the experiment. This results in the agents and the

biofilm as a whole experiencing the appropriate drag forces they would feel in the experiment while also exiting the simulation roughly 100 times faster once they have detached. This does however lead to inaccuracies in the agent momentum which could influence agents colliding into other sections of the biofilm.

6 Sensitivity of Distance Threshold for Categorising Events

For this study, the distance-threshold clustering algorithm created to group detachment events used a value of 0.05 μm between two agent surfaces as the threshold for whether the two agents are classified as part of the same event. This section presents a sensitivity analysis to observe how the results change when varying this threshold value. The test case used for this analysis was the biofilm with 30% EPS production fraction and no QS capabilities in a flow speed of 1.86 mm/s. Supplementary figure 3 shows the event frequency distribution for the test case with a distance threshold of 0.01 μm, 0.05 μm, 0.1 μm, 1 μm and 2 μm. The result remains unchanged until the distance threshold increases above 1 μm, at which point small events (1 - 3 cells) are less represented and medium events (4 - 100 cells) are more represented. Large events (100 cells) remain unchanged.

7 Supplementary Analysis

This section includes further analysis of the cell detachment rates. Supplementary figure 4 shows the number of detached cells per timestep for biofilms with EPS production fraction of 20%, 30%, 40% and 50% in a flow speed of 1.86 mm/s. As the EPS production fraction increases, the peaks (sloughing events) become larger and less frequent. The position of the baseline (erosion rate) also decreases. This further illustrates the prediction that as the fraction of EPS in the biofilm increases, cells become more likely to detach via sloughing than erosion. Supplementary figure 5 shows the number of detached cells per timestep for biofilms with EPS production fraction of 30% in a flow speed of 1 mm/s, 2 mm/s, 3 mm/s and 4 mm/s. As the flow speed increases, the peaks decrease in height but roughly stay the same frequency. The baseline also remains unchanged. Supplementary figure 6 shows the number of detached cells per timestep for biofilms with EPS production fraction of 30% with and without QS in a flow speed of 1.86 mm/s. When including QS, spikes become less frequent and the baseline is slightly raised. However, the size of the spikes remain the same.

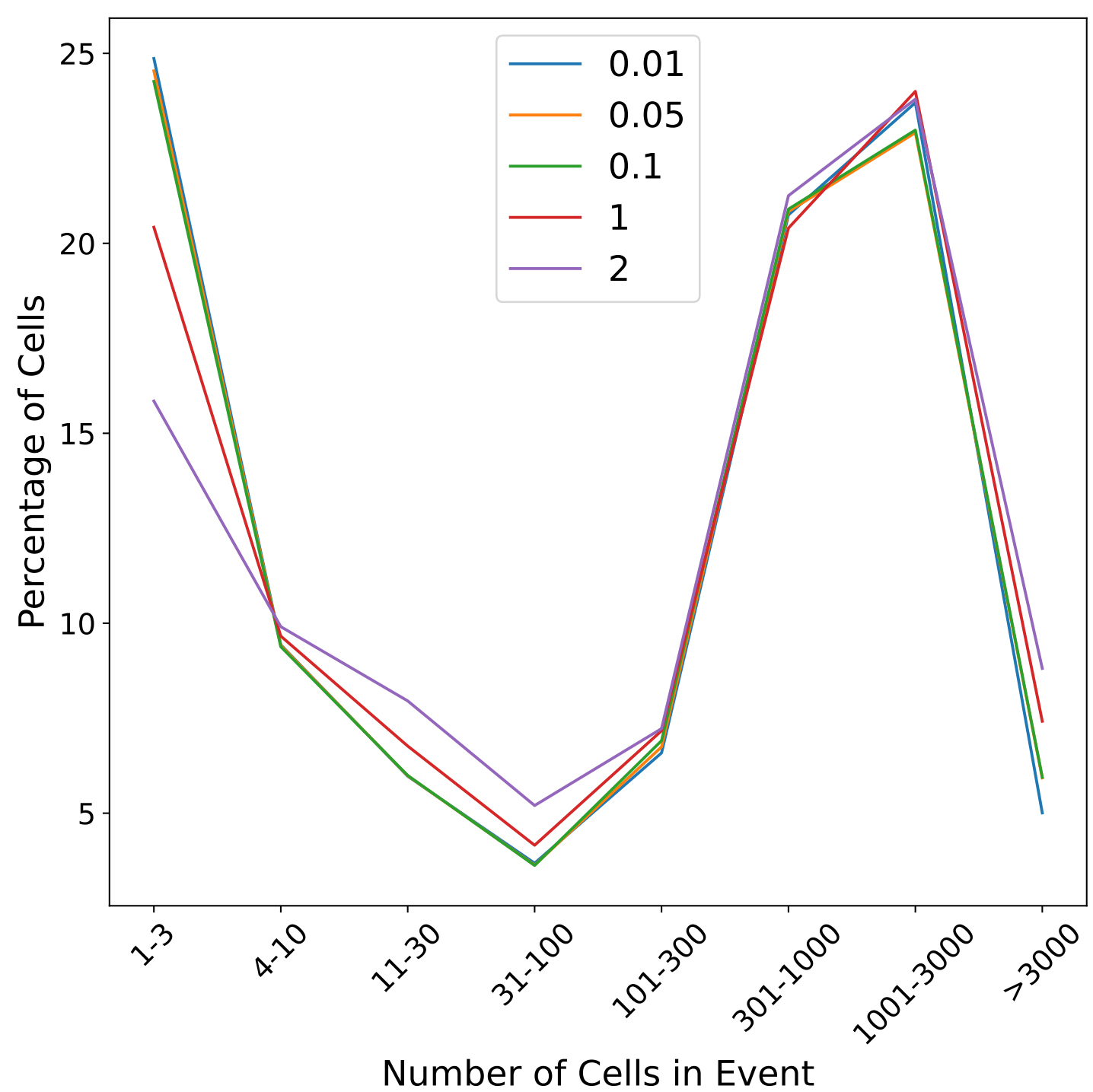

Supplementary Figure 3: **Sensitivity analysis for the distance-threshold clustering algorithm.** Small events become less represented as the threshold for distance between two agent surfaces increases.

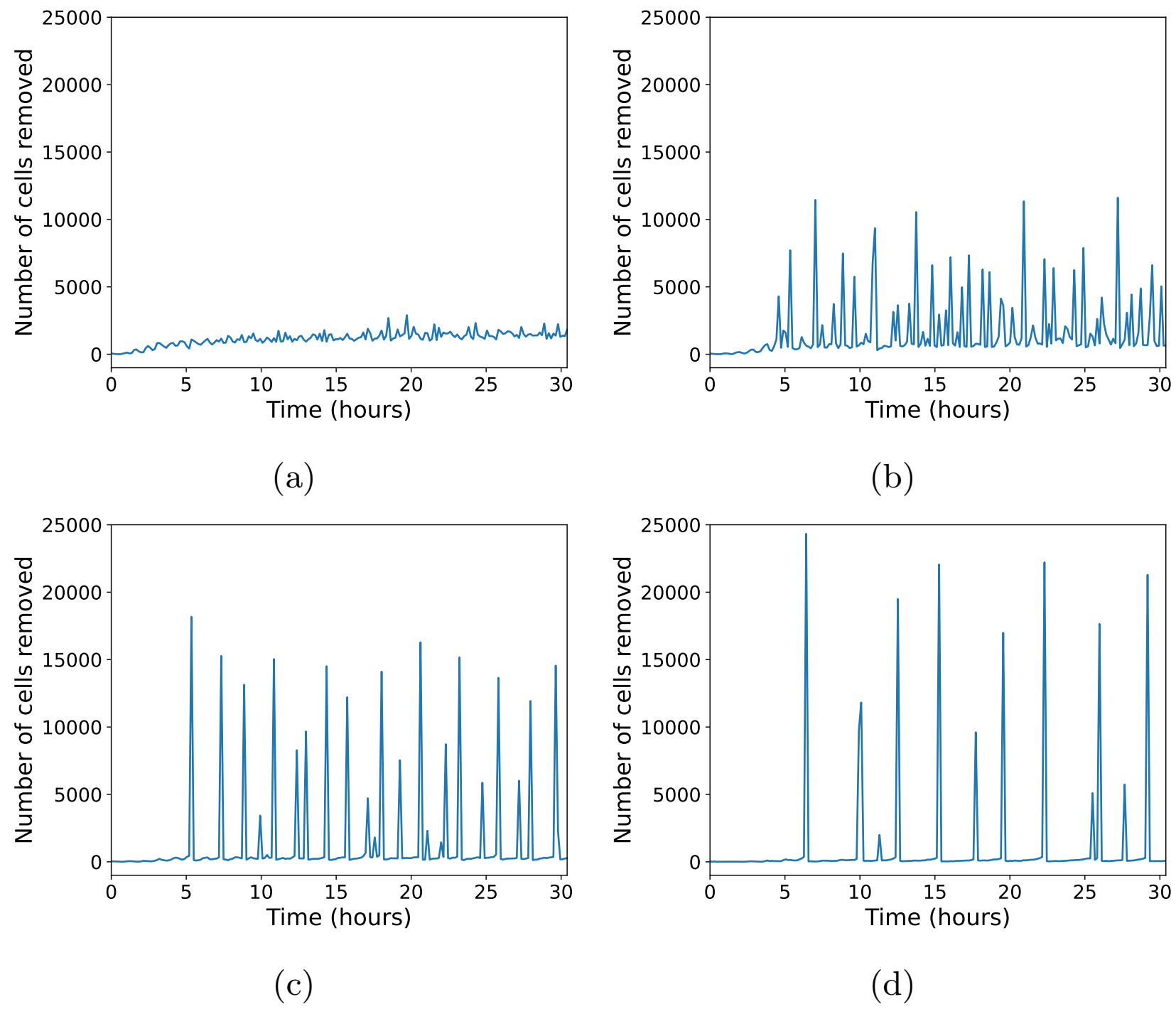

Supplementary Figure 4: **Influence of EPS production fraction on number of cells detached per timestep.** Simulated biofilms with (a) 20%, (b) 30%, (c) 40% and (d) 50% EPS production fraction in a flow speed of 1.86 mm/s. Spikes in the number of detached cells indicate sloughing events and the baseline indicate erosion rate.

Supplementary Movies

Supplementary Movie 1: Biofilm simulation at 30% EPS production and 1.86 mm/s flow speed.

Supplementary Movie 2: Sloughing event at 22.9 hours of growth.

Supplementary Movie 3: Biofilm simulation at 20% EPS production and 1.86 mm/s flow speed.

Supplementary Movie 4: Biofilm simulation at 50% EPS production and 1.86 mm/s flow speed.

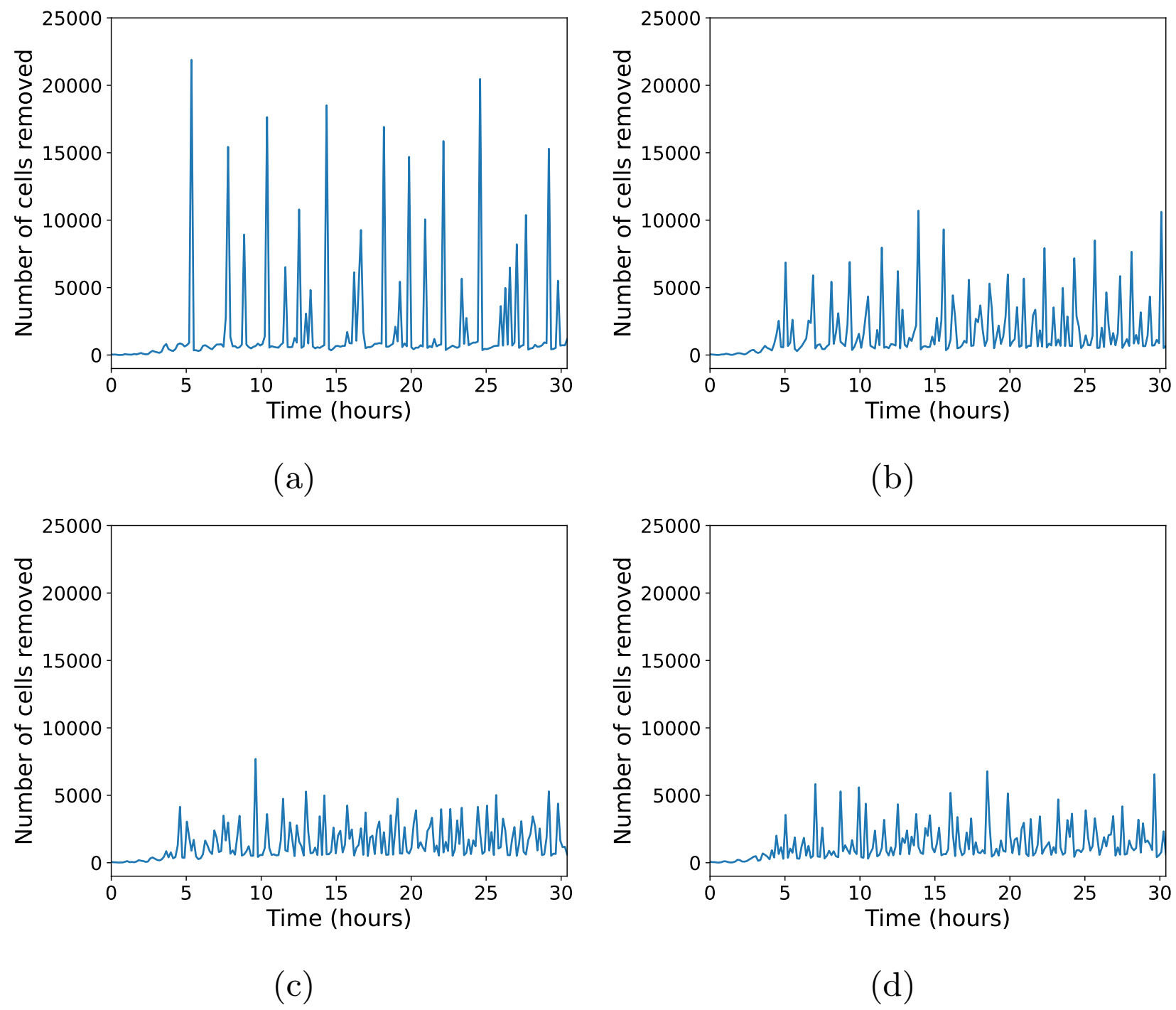

Supplementary Figure 5: **Influence of flow speed on number of cells detached per timestep.** Simulated biofilms with 30% EPS production fraction in flow speeds of (a) 1 mm/s, (b) 2 mm/s, (c) 3 mm/s and (d) 4 mm/s. Spikes in the number of detached cells indicate sloughing events and the baseline indicate erosion rate.

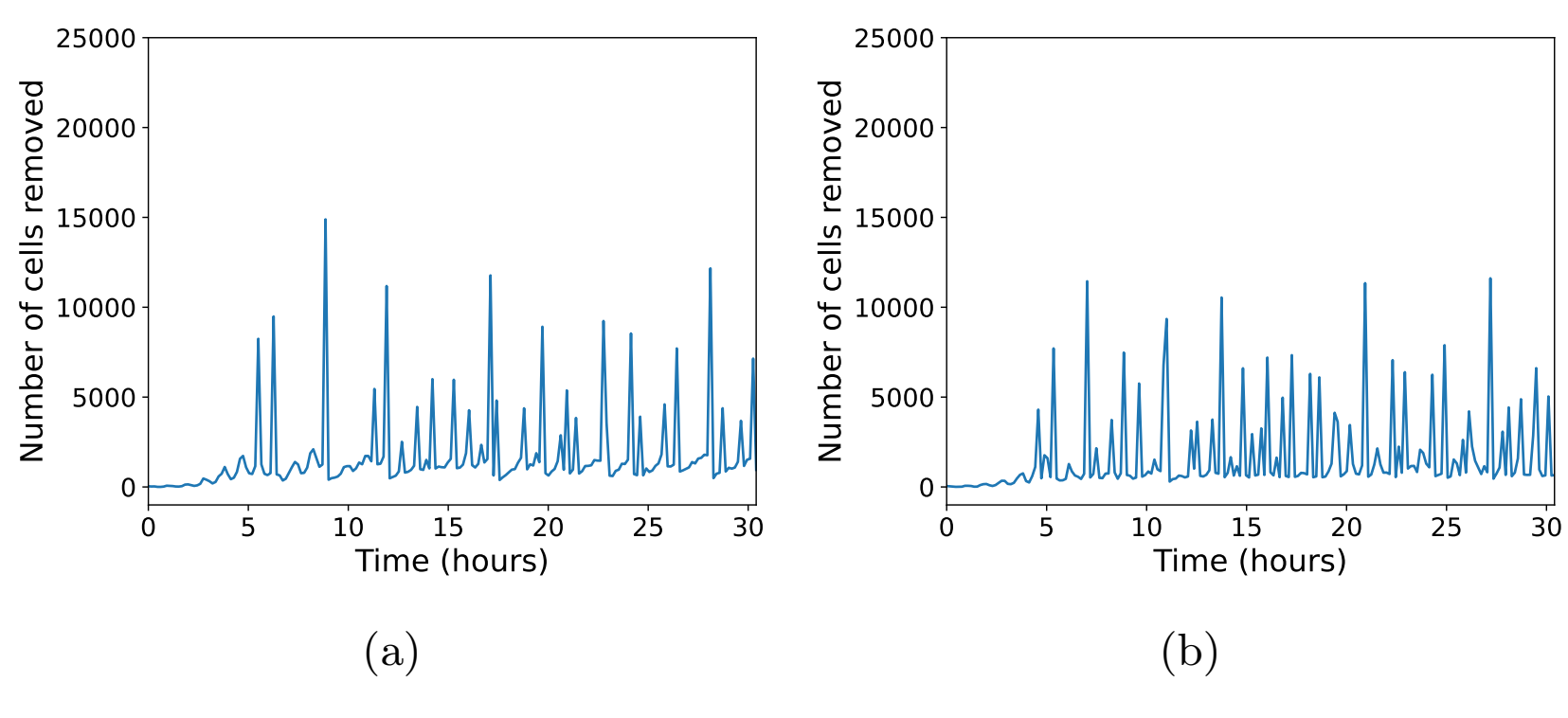

Supplementary Figure 6: **Influence of quorum-sensing on number of cells detached per timestep.** (a) simulated biofilms capable of QS and (b) simulated biofilms incapable of QS with 30% EPS production fraction in a flow speed of 1.86 mm/s. Spikes in the number of detached cells indicate sloughing events and the baseline indicate erosion rate.

References

[1] Stephen P Diggle and Marvin Whiteley, (2020) **Microbe Profile: Pseudomonas aeruginosa: opportunistic pathogen and lab rat**, *Microbiology*: 30-33, doi:10.1099/mic.0.000860

[2] Annette E. LaBauve and Matthew J. Wargo, (2012) **Growth and laboratory maintenance of Pseudomonas aeruginosa**, *Current protocols in microbiology* 25.1: 6E-1, doi:10.1002/9780471729259.mc06e01s25

[3] Pahala Gedara Jayathilake, et al, (2017) **A mechanistic Individual-based Model of microbial communities**, *PloS one* 12.8: e0181965, doi:10.1371/journal.pone.0181965

[4] Yuqing Xia, et al, (2022) **Coupled CFD-DEM modeling to predict how EPS affects bacterial biofilm deformation, recovery and detachment under flow conditions**, *Biotechnology and Bioengineering* 119.9: 2551-2563, doi:10.1002/bit.28146

[5] Suzanne Wilson, et al, (2004) **Statistical quantification of detachment rates and size distributions of cell clumps from wild-type (PAO1) and cell signaling mutant (JP1) Pseudomonas aeruginosa biofilms**, *Applied and environmental microbiology* 70.10: 5847-5852, doi:10.1128/AEM.70.10.5847-5852.2004

[6] Gaurav Kathuria, Aditya Karnik and Mohit Tandon, (2015) **Study of gas-solid fluidized bed - tuning Syamlal O'Brien drag model**, technical report from CD-adapco, doi: 10.13140/RG
